# Supplementary material for: VATS Pleurectomy Decortication Is a Reasonable Alternative for Higher Risk Patients in the Management of Malignant Pleural Mesothelioma: An Analysis of Short-Term Outcomes
Source: Cancers (Basel). 2021 Mar 3;13(5):1068. doi: 10.3390/cancers13051068 (PMC7959123; doi:10.3390/cancers13051068)
Supplement: Supplementary file 1 [file cancers-13-01068-s001.pdf]

cancers MDPI

Supplementary Materials

VATS Pleurectomy Decortication is a Reasonable Alternative for Higher Risk Patients in the Management of Malignant Pleural Mesothelioma: an Analysis of Short Term Outcomes

Dong-Seok Lee *, Andrea Carollo, Naomi Alpert, Emanuela Taioli and Raja Flores

Table 1. Diagnosis and Procedure Codes to Identify Surgical Pleural Mesothelioma Patients.

	**Pleural Mesothelioma Diagnosis Codes**	
	ICD-9 Diagnosis Codes*	*ICD-10* Diagnosis Codes*
	163, 163.0, 163.1, 163.8, 163.9	C45.0, C38.4
	**Mesothelioma Surgery Procedure Codes**	
	ICD-9 Procedure Codes*	*ICD-10* Procedure Codes*
Minimally Invasive PD	345.2	0BDN3ZZ, 0BDP3ZZ, 0BDN4ZZ, 0BDP4ZZ, 0BBN3ZZ, 0BBN4ZZ, 0BBN8ZZ, 0BBP3ZZ, 0BBP4ZZ, 0BBP8ZZ
Open PD	345, 345.1	0BDN0ZZ, 0BDP0ZZ, 0BBN0ZZ, 0BBP0ZZ
Minimally Invasive EPP	325.0	0BTK4ZZ, 0BTL4ZZ, 0BTM4ZZ
Open EPP	325, 325.9	0BTK0ZZ, 0BTL0ZZ, 0BTM0ZZ

*The change from ICD-9 to ICD-10 codes was effective October, 1, 2015
Abbreviations: PD, Pleurectomy Decortication; EPP, Extrapleural pneumonectomy

Table 2. Diagnosis Codes^ to Identify Complications.

Complication	ICD-9 Diagnosis Code	ICD-10 Diagnosis Code
Cardiovascular		
Supraventricular Arrhythmia	427.0, 427.3, 427.31, 427.32	I48, I47.1
Myocardial Infarction	410, 411.81, 413	I21, I24.0, I20
Postoperative Stroke	997.02	I97.811, I97.821
Deep Venous Thromboembolism	451.1, 451.2, 451.81, 451.9, 453.2, 453.40-453.42, 453.8, 453.9,	I80.1, I80.2, I80.30, I80.9, I82.220, I82.210, I82.4 I82.6, I82.290, I82.890, I82.90, I82.A1, I82.B1, I82.C1
Pulmonary Embolism	415.1, 415.11, 415.12, 415.19	I26.9
Pulmonary		
Pneumonia	486, 481, 482.0-482.3, 482.41, 482.49, 482.8, 482.9, 997.31	J13, J14, J15.0, J15.1, J15.211, J15.29, J15.3-J15.9, J18, J95,851
Postoperative acute respiratory insufficiency	518.5	J95.1, J95.821, J95.822, J96
Postoperative acute pneumothorax	512.1	J95.811, J95.812
Postoperative pulmonary edema	518.4	J81.0
Pulmonary collapse	518.0	J98.11, J98.19
Empyema	510.0, 510.9	J86.0, J86.9
Mechanical ventilation*	96.70, 96.71, 96.72, 93.90	5A1935Z, 5A1945Z, 5A1955Z, 5A09357, 5A09457, 5A09557
Infection		
Sepsis/shock	038, 995.91, 995.92, 998.0, 999.3	A40, A41, T81.1, T81.44,
Urinary Tract Infection	599.0, 590.9	N39.0, N15.9
Postoperative Infection	998.51, 998.59	T81.40, T81.41, T81.42, T81.43, T81.49

Intraoperative		
Puncture/Laceration	998.2	I97.51, I97.52, J95.71, J95.72
Bleeding	998.11	I97.418, I97.42, J95.61, J95.62

*Procedure Codes

^The change from ICD-9 to ICD-10 codes was effective October, 1, 2015
